# Expression of signaling adaptor proteins predicts poor prognosis in pancreatic ductal adenocarcinoma

**DOI:** 10.1186/s13000-017-0633-4

**Published:** 2017-05-30

**Authors:** Lili Wang, Junliang Lu, Huanwen Wu, Li Wang, Xiaolong Liang, Zhiyong Liang, Tonghua Liu

**Affiliations:** Molecular Pathology Research Center, Department of Pathology, Peking Union Medical College Hospital, Chinese Academy of Medical Sciences and Peking Union Medical College, Beijing, 100730 China

**Keywords:** Pancreatic ductal adenocarcinoma, GAB, CRKL, FRS2, Prognosis

## Abstract

**Background:**

Adaptor proteins bridge the gap between cell surface receptors and their downstream signaling elements. The clinicopathological and prognostic values of adaptor proteins remain poorly understood. The purpose of the present study was to explore the expression and prognostic value of three adaptor proteins: GRB2-associated binding protein 2 (GAB2), CRK-like protein (CRKL) and fibroblast growth factor receptor substrate 2 (FRS2) in pancreatic ductal adenocarcinoma (PDAC).

**Methods:**

The expression of GAB2, CRKL, and FRS2 in 77 formalin fixed paraffin embedded (FFPE) samples from 77 PDAC patients, along with three paired fresh PDAC and matched normal tissues from 3 PDAC patients was analyzed by immunohistochemistry and western blot, respectively. The association between the expression of the three proteins and the clinicopathological factors of PDAC was assessed by *χ* ^2^ test. The correlation between the expression levels of the three proteins was analyzed by Spearman rank correlation analyses; Kaplan-Meier survival analyses were also performed.

**Results:**

IHC was successful in 75, 76, and 77 cases for GAB2, CRKL, and FRS2, respectively. Of which, the positive rate of GAB2, CRKL, and FRS2 protein expression was 40.00% (30/75), 53.95% (41/76) and 35.06% (27/77), respectively. The positive rate of GAB2, CRKL and FRS2 co-expression was 16.88% (13/77). Though there was no association between GAB2 expression, CRKL expression, FRS2 expression, GAB2/CRKL/FRS2 co-expression and the clinicopathological parameters of PDAC, positive correlations were observed between the expressions of the three proteins. Further, univariate survival analysis showed that positive expression of GAB2, CRKL and FRS2 and co-expression of GAB2/CRKL/FRS2 of PDAC predicted poor clinical outcomes, and multivariate survival analysis suggested that positive expression of GAB2 and positive co-expression of GAB2/CRKL/FRS2 were independent prognostic factors for disease-free survival (DFS) and overall survival (OS), respectively.

**Conclusion:**

In conclusion, GAB2, CRKL, and FRS2 may be potential prognosticators and therapeutic targets for PDAC patients.

## Background

Aberrant activation of receptor tyrosine kinases (RTKs) and downstream signaling pathways is ubiquitous in tumor cells, which contributes to the genesis and progression of various types of cancers [1]. Therefore, RKTs signal pathways have become primary targets for cancer therapy [2].

Adaptor proteins are the bridging elements that connect the membrane-docking RTKs and downstream signal components in the pathways. They facilitate key signaling transduction events, regulate signal specificity and amplification by providing an essential scaffolding function to recruit signal molecules into signaling networks [3]. Based on the phosphorylation ability, adaptor proteins are divided into two groups. The first group of adaptor proteins has phosphorylation sites and some feature a membrane docking domain. Group members include GRB2-associated binding protein (GAB), fibroblast growth factor receptor substrate 2 (FRS2), insulin receptor substrate (IRS), Src homology 2-containing protein (SHC), and downstream of the kinase (DOK)-family proteins. The second group comprises GRB2, CRKL, and NCK [4], on which phosphorylation site is absent. Previous studies have demonstrated that adaptor protein-encoding genes are amplified in various human cancers and considered potential oncogenes, promising prognosticators and therapeutic targets [5–9].

GAB2, CRKL, and FRS2 are three adaptor proteins that exert important roles in signaling transduction of RTKs [10]. Besides, they have been found to participate in the genesis and progression of various cancers including lung adenocarcinoma, ovarian cancer and breast cancer [10]. Two earlier studies reported that GAB2 and CRKL were overexpressed in pancreatic ductal adenocarcinoma (PDAC), but they did not investigate the association of GAB2 and CRKL overexpression with the prognosis of PDAC patients [11, 12]. Also, the clinical significance of FRS2 expression level in PDAC is unrevealed.

The aim of the present study was to explore the expression level of GAB2, FRS2, and CRKL in PDAC and test if there is a relationship between their expressions and the clinicopathological characteristics as well as the prognosis of PDAC.

## Methods

### Patients and clinicopathological data

FFPE samples from 77 PDAC patients and fresh samples of three PDAC patients from the pathology department of Peking Union Medical College Hospital between January 2011 and January 2016 were included in the present study according to the following inclusion criteria. 1) PDAC patients without preoperative adjuvant therapy. 2) PDAC patients with complete tumor resection surgery. 3) PDAC patients with complete clinicopathological and follow-up data. 4) Hematoxylin and eosin-stained slides of all samples were reconfirmed by two experienced histopathologists (WLL and WHW). The FFPE specimens were preserved at room temperature, and fresh samples were stored at −80 °C.

### Tissue Microarray (TMA) construction

H&E slides for each FFPE tissue block were reviewed, and representative tumor and adjacent normal regions were marked on the blocks. Paired cancer and normal cores were punched and transferred to a recipient block to make the TMA block. For each patient, one tumor core and one normal core were taken from the donor block.

### Immunohistochemistry staining

Immunohistochemistry staining was performed as previously described [13]. Briefly, 4-μm-thick TMA slides were baked for one hour at 60 °C, deparaffinized, dehydrated and treated in citrate buffer (pH 6.0). The TMA slides were then incubated with 3% hydrogen peroxide at room temperature for one hour, followed by incubation of anti-GAB2 (ab108423, 1:25 dilution, Abcam, Cambridge, UK), CRKL (ab32126; 1:50 dilution; Abcam, Cambridge, UK), and FRS2 (AF4069; 1:25 dilution; R&D, Minneapolis, USA) antibodies at 4 °C overnight. The second day, the slides were incubated with secondary antibodies (Pre-diluted; Zhongshan Golden Bridge, Beijing, China), stained, counterstained, dehydrated, cleared, and mounted. Positive and negative controls were included.

### Evaluation of immunohistochemical staining

Staining intensity was assigned to scores 0, 1, 2, 3 representing “no staining,” “weak staining,” “moderate staining,” and “strong staining,” respectively. The percentage of positive cancer cells was divided into four bands: “0%”, “1–25%”, “25–50%”, and “>50%” and was assign to scores of 0 to 3, respectively. By the semi-quantitative immunoreactive score (IRS) system, the final scores of GAB2, CRKL, and FRS2 were calculated according to a general scheme described in [14]. Briefly, after multiplying the staining intensity score by the staining percentage score, a final score >2 was considered GAB2 positive; Final score >0 was considered CRKL positive and final score >4 was considered FRS2 positive. Co-expression of GAB2/CRKL/FRS2 was defined as the positivity of all three immunomarkers at the same time. The slide was evaluated by two histopathologists, where discrepancies were solved by discussion.

### Western blot analysis

Tissue protein lysate was obtained by liquid nitrogen grounding, followed by RIPA buffer digestion and centrifugation. 30 μg tissue lysate per lane was loaded onto 10% sodium dodecyl sulfate (SDS) polyacrylamide gels for electrophoresis, and protein was then transferred to Polyvinylidene difluoride (PVDF) membranes, incubated with primary polyclonal anti-GAB2 (ab108423; 1:1000 dilution; Abcam, Cambridge, UK), CRKL (ab32126; 1:1000 dilution; Abcam, Cambridge, UK), and FRS2 (AF4069; 1:1000 dilution; R&D, Minneapolis, USA) antibodies, respectively. Followed by incubation with the horseradish peroxidase (HRP)—conjugated secondary antibody (1:10000 dilution; Zhongshan Golden Bridge, Beijing, China). The EMD Millipore Immobilon™ Western Chemiluminescent HRP Substrate (Millipore, Darmstadt, Germany) was then applied according to the manufacturer’s instructions. Documentation of the chemiluminescence was achieved by exposure on a Carestream X-OMAT BT X-ray film (Carestream, Xiamen, China). The latter was then scanned by a Microtek ScanMaker i700 scanner (Microtek Inc., Shanghai, China).

### Statistical analysis

SPSS 17.0 was used to perform the statistical analyses (SPSS Inc., Chicago, IL). The associations between the three proteins and other clinicopathological factor were assessed by *χ* ^2^ test. Kaplan-Meier method and log-rank test were used for survival analysis. Spearman rank correlation analysis was used to analyze the correlation between all the three indexes. The influence of various factors on survival was analyzed individually by univariate survival analysis. Variables with *p* < 0.05 in the univariate analysis were further analyzed using multivariate analysis. A *p* < 0.05 was considered statistically significant.

## Results

### GAB2, FRS2, and CRKL were frequently expressed in PDAC tissues

**Fig. 1:**
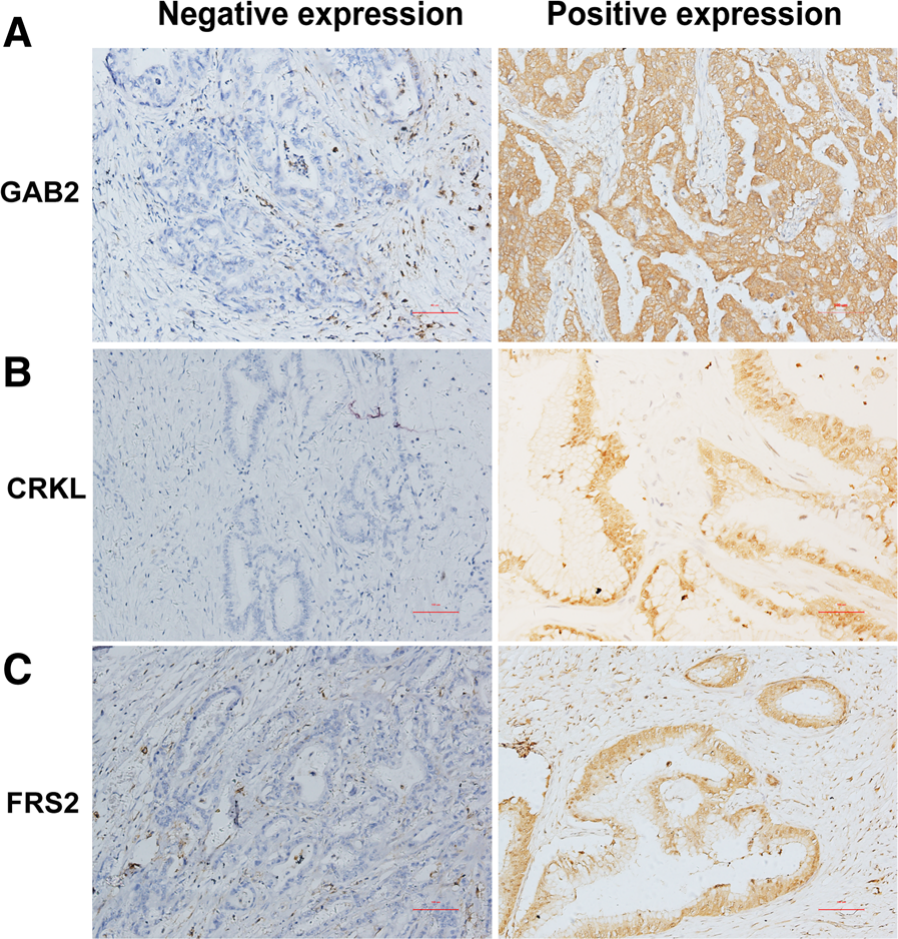
Expression of GAB2, CRKL and FRS2: GAB−/+ expression (**a**) CRKL−/+ expression (**b**) FRS2−/+ expression (**c**). Magnification × 100

**Fig. 2:**
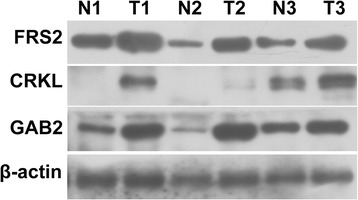
Western blot analyses of GAB2, CRKL and FRS2 expression in PDAC and matched normal tissues. T: PDAC tissue; N: peritumor normal tissue. β-actin was used as a loading control

IHC was successful on 75, 76, and 77 cases for GAB2, CRKL, and FRS2, respectively. Failure was attributed to the tissue torn-off from the slides. The demographic characteristics of the 77 patients are shown in Table 1. The positive rates in tumor regions for GAB2, CRKL, and FRS2 were 40.00% (30/75), 53.95% (41/76), and 35.06% (27/77), respectively. Staining patterns of anti-GAB2 and anti-CRKL antibodies were both cytoplasmic and nuclear in the tumor regions, while that of the anti-FRS2 antibody was cytoplasmic and membranous in the tumor regions. GAB2, CRKL, and FRS2 were also expressed in the mesenchymal cells and the infiltrating inflammatory cells (Fig. 1). Moreover, none of the three proteins were expressed in the normal acinar or ductal epithelial cells. However, they were positive in the pancreatic islets (data not shown). Similarly, Western blot analysis revealed higher levels of GAB2, CRKL, and FRS2 proteins were expressed in PDAC tissues than in the matched adjacent normal tissues (Fig. 2).

**Table 1:** Clinical and pathological characteristics of PDCA patients

Parameter	No. of patients (%) Number(%)
Sex	
Female	36 (46.75%)
Male	41 (53.25%)
Age at diagnosis	
≤ 60	37 (48.05%)
> 60	40 (51.95%)
Size (diameter), cm	
≤ 2	16 (20.78%)
> 2	61 (79.22%)
Tumor sites	
Head	40 (51.95%)
Body/Tail	37 (48.05%)
Resection margins	
Negative	64 (83.12%)
Positive	13 (16.88%)
Differentiation	
Well/moderate	58 (75.32%)
Poor	19 (25.68%)
Nodal metastasis	
No	30 (38.96%)
Yes	47 (61.04%)
TNM stage	
I/II	65 (84.42%)
III/IV	12 (15.58%)

### Correlations among GAB2, FRS2, and CRKL expression

**Fig. 3:**
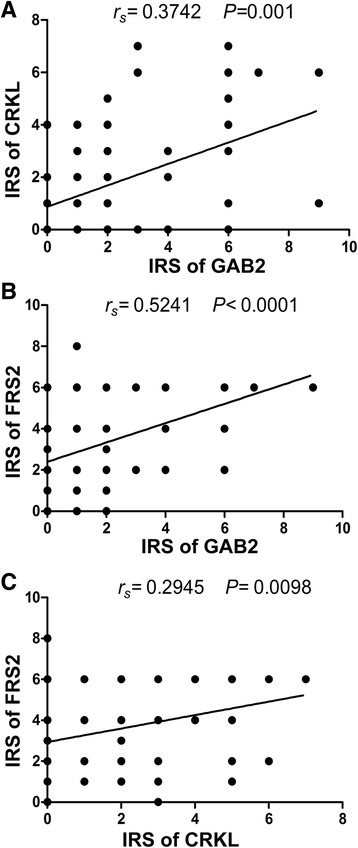
Correlations between GAB2 and CRKL expression, GAB2 and FRS2 expression, and CRKL and FRS2 expression in PDAC: GAB2 expression correlated with CRKL (*r* = 0.3742, *P* = 0.001) (**a**) GAB2 expression correlated with FRS2 (*r* = 0.5241, *P* < 0.001) (**b**) CRKL expression correlated with FRS2 (*r* = 0.2945, *P* = 0.0098) (**c**)

Spearman’s rank correlation analysis showed that there was a moderately positive correlation between the expression of GAB2 and CRKL (*r* = 0.3742, *P* = 0.001), and a moderately positive correlation between the expression of GAB2 and FRS2 (*r* = 0.5241, *P* < 0.001). The expression of CRKL was weakly but positively correlated with FRS2 (*r* = 0.2945, *P* = 0.0098) (Fig. 3).

### There was no association between the expression of GAB2, CRKL, FRS2 and other clinicopathologic parameters of PDAC

No significant association was observed between the expression of GAB2, CRKL, FRS2, or the co-expression of GAB/CRKL/FRS2 and the clinicopathological parameters of PDAC patients (Table 2).

**Table 2:** Correlation between GAB2/CRKL/FRS2 expression and clinicopathological characteristics of PDCA

Parameter	GAB2 n (%)		*P*	CRKL n (%)		*P*	FRS2 n (%)		*P*	GAB2/CRKL/FRS2 n (%)		*P*
	Negative	Positive		Negative	Positive		Negative	Positive		Negative^a^	Positive^b^	
Sex			1.000			1.000			1.000			0.762
Female	22(61.1%)	14(38.9%)		17(47.2%)	19(52.8%)		23(63.9%)	13(36.1%)		29(80.6%)	7(19.4%)	
Male	23(59.0%)	16(41.0%)		18(45.0%)	22(55.0%)		27(65.9%)	14(34.1%)		35(85.4%)	6(14.6%)	
Age at diagnosis			1.000			0.645			0.811			1.000
≤ 60	21(60.0%)	14(40.0%)		18(50.0%)	18(50.0%)		25(67.6%)	12(32.4%)		31(83.6%)	6(16.2%)	
> 60	24(60.0%)	16(40.0%)		17(42.5%)	23(57.5%)		25(62.5%)	15(37.5%)		33(82.5%)	7(17.5%)	
Size (diameter),			0.255			0.260			0.557			0.452
≤ 2 cm	7(46.7%)	8(53.3%)		5(31.3%)	11(68.7%)		9(56.3%)	7(43.8%)		12(75.0%)	4(25.0%)	
> 2 cm	38(63.3%)	22(36.7%)		30(50.0%)	30(50.0%)		41(67.2%)	20(32.8%)		52(85.2%)	9(14.8%)	
Tumor sites			0.246			0.645			0.161			0.367
Head	26(66.7%)	13(33.3%)		17(42.5%)	23(57.5%)		29(72.5%)	11(27.5%)		35(87.5%)	5(12.5%)	
Body/Tail	19(52.8%)	17(47.2%)		18(50.0%)	18(50.0%)		21(56.8%)	16(43.2%)		29(78.4%)	8(21.6%)	
Resection margins			1.000			1.000			1.000			1.000
Negative	37(59.7%)	25(40.3%)		29(46.0%)	34(54.0%)		41(64.1%)	23(35.9%)		53(82.8%)	11(17.2%)	
Positive	8(61.5%)	5(38.5%)		6(46.2%)	7(53.8%)		9(69.2%)	4(30.8%)		11(84.6%)	2(15.4%)	
Differentiation			1.000			0.187			0.581			0.288
Well/moderate	34(60.7%)	22(39.3%)		29(50.9%)	28(49.1%)		39(67.2%)	19(32.8%)		50(86.2%)	8(13.8%)	
Poor	11(57.9%)	8(42.1%)		6(31.6%)	13(68.4%)		11(57.9%)	8(42.1%)		14(73.7%)	5(26.3%)	
Nodal metastasis			0.640			0.642			0.337			0.122
No	17(56.7%)	13(43.3%)		13(41.9%)	18(58.1%)		18(58.1%)	13(41.9%)		23(74.2%)	8(25.8%)	
Yes	28(62.2%)	17(37.8%)		22(48.9%)	23(51.5%)		32(69.6%)	14(30.4%)		41(89.1%)	5(10.9%)	
TNM stage			1.000			1.000			1.000			1.000
I/II	38(60.3%)	25(39.7%)		30(46.2%)	35(53.8%)		42(64.6%)	23(35.4%)		54(83.1%)	11(16.9%)	
III/IV	7(58.3%)	5(41.7%)		5(45.5%)	6(54.5%)		8(66.7%)	4(33.3%)		10(83.3%)	2(16.7%)	

^a^Negative indicates that one or two of the indexes: GAB2, CRKL, and FRS2 are positive. The three torn-off specimens happened to be from tumors that are negative for one or two of the three proteins and are thus categorized into the GAB2/CRKL/FRS2 negative group despite not knowing the actual expression of one or two of the adaptor proteins

^b^Positive indicates all indexes positive of GAB2,CRKL and FRS2

### Expression of GAB2, CRKL, FRS2 and co-expression of GAB2 /CRKL/FRS2 were predictors for poor prognosis of PDAC patients

**Fig. 4:**
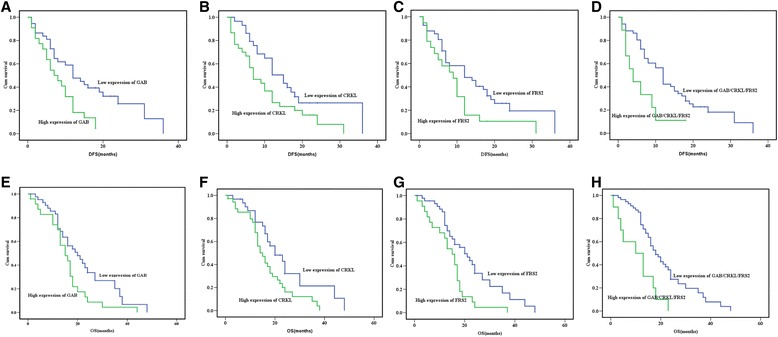
Kaplan-Meier curves of DFS and OS with different expression patterns of GAB2, CRKL, and FRS2. DFS according to GAB2 expression pattern (*P* = 0.005) (**a**) CRKL expression pattern (*P* = 0.036) (**b**) FRS2 expression pattern (*P* = 0.037) (**c**) and GAB2/CRKL/FRS2 co-expression pattern (*P* = 0.015) (**d**). OS according to GAB2 expression pattern (*P* = 0.031) (**e**) CRKL expression pattern (*P* = 0.020) (**f**), FRS2 expression pattern (*P* = 0.003) (**g**) and GAB2/CRKL/FRS2 co-expression pattern (*P* = 0.001) (**h**)

The follow-up time of the enrolled patients in our study was between 1 to 5 years. The median DFS was 10.0 months, and the median OS was 16.0 months. In addition to poor tumor differentiation, lymph node metastasis, resection margin, and advanced TNM stage, univariate survival analyses revealed that the expression of GAB2, CRKL, FRS2 and the co-expression of GAB2/CRKL/FRS2 were also indicators for poor clinical outcome. Furthermore, patients co-expressing GAB/CRKL/FRS2 in the tumor had a significantly worse outcome than those with single marker expression (Table 3, Fig. 4). In the multivariate analysis, poorer tumor differentiation and more advanced TNM stage were independent indicators for unfavorable prognosis regarding both DFS and OS (Table 4), which served as an indicator for the representativeness of the present dataset. Further, multivariate analysis revealed that positive expression of GAB2 increased the hazard ratio of recurrence (PFS), while positive co-expression of GAB2/CRKL/FRS2 increased the hazard ratio of death (OS).

**Table 3:** Univariate analyses for DFS and OS

Variable	DFS		OS	
	Median survival(months)	*P*	Median survival(months)	*P*
Sex		0.700		0.506
Female	10.00		17.00	
Male	12.00		17.00	
Age at diagnosis		0.725		0.212
≤ 60	12.00		17.00	
> 60	10.00		18.00	
Size (diameter),		0.300		0.493
≤ 2 cm	14.00		18.00	
> 2 cm	10.00		16.00	
Tumor sites		0.480		0.910
Head	10.00		17.00	
Body/Tail	12.00		17.00	
Resection margins		0.265		**0.013**
Negative	12.00		18.00	
Positive	7.00		13.00	
Differentiation		**0.024**		**0.004**
Well/moderate	12.00		19.00	
Poor	7.00		13.00	
Nodal metastasis		0.394		**0.076**
No	12.00		19.00	
Yes	8.00		16.00	
TNM stage		**0.001**		**<0.001**
I/II	12.00		18.00	
III/IV	5.00		9.00	
GAB2 expression		**0.005**		**0.031**
Negative	12.00		20.00	
Positive	7.00		15.00	
CRKL expression		**0.036**		**0.020**
Negative	15.00		20.00	
Positive	7.00		15.00	
FRS2 expression		**0.037**		**0.003**
Negative	12.00		21.00	
Positive	9.00		15.00	
GAB2/CRKL/FRS2 expression		**0.015**		**0.001**
Negative	12.00		18.00	
Positive	4.00		10.00	

Bold values indicate statistical significance

**Table 4:** Multivariate analyses for DFS and OS

Parameter	HR(95%CI)	*P*
DFS: Cox regression model		
Differentiation		**0.033**
Well/moderate		
Poor	2.062(1.059–4.016)	
TNM stage		**0.004**
I/II		
III/IV	3.180(1.459–6.928)	
GAB2 expression		**0.018**
Negative		
Positive	2.118(1.140–3.936)	
OS: Cox regression model		
Differentiation		**0.043**
Well/moderate		
Poor	2.235(1.129–4.422)	
TNM stage		**<0.001**
I/II		
III/IV	7.416(3.052–18.022)	
GAB2/CRKL/FRS2 coexpression		**0.013**
Negative		
Positive	2.642(1.226–5.693)	

Bold values indicate statistical significance

## Discussion

With the development of the inhibition strategies for protein-protein interactions and the emerging disadvantages of traditional targeting therapies, adaptor proteins are becoming promising targets for the therapy of human cancers [15]. The present study investigated the expression, clinical and prognostic significance of GAB2, CRKL and FRS2 in 77 PDAC patients. Our data demonstrated that GAB2, CRKL, and FRS2 were frequently expressed in PDAC. More importantly, the present study revealed that expression of GAB2, CRKL, FRS2, and the co-expression of GAB2/CRKL/FRS2 predicted poor prognosis for PDAC patients. Finally, expression of GAB2 and co-expression of GAB2/CRKL/FRS2 were independent prognostic factors regarding DFS and OS, respectively.

GAB2 is a key member of adaptor protein family and plays an important role in the tumorigenesis and progression of various human cancers [10]. A previous study revealed that GAB2 is expressed in PDAC patients by the reverse-phase protein assay (RPPA) [11]. In the present study, we not only confirmed that GAB2 was expressed in PDAC patients by IHC, but also demonstrated for the first time that GAB2 was associated with a poor outcome of the PDAC patients and that it served as an independent prognostic factor regarding DFS. While several studies indicated that GAB2 was associated with tumor metastasis in breast cancer, colorectal cancer, melanoma, and ovarian cancer [16–19], our study failed to reveal a significant relationship between GAB2 expression and lymph node metastasis in PDAC patients. Our finding indicated that GAB2 might have conferred poorer prognoses for PDAC patients via mechanisms other than facilitating lymph node metastases and further investigation is warranted.

CRKL gene amplification and high levels of protein expression were reported in many human cancers [10], suggesting that CRKL may act as an oncogene. A previous study demonstrated that high expression of CRKL promoted proliferation and invasion of pancreatic cancer cells, but did not look into the clinicopathological association or prognostic values of this marker [12]. The present study demonstrated for the first time that high expression of CRKL was associated with a poor prognosis of PDAC, but not with other well-established clinicopathological factors, including lymph node metastasis.

As another member of adaptor proteins, FRS2 was amplified in various human cancers, including ovarian cancer, liposarcoma and glioma [6, 20, 21]. FRS2 predominantly plays its scaffolding function by regulating key signaling pathways downstream of fibroblast growth factor receptors (FGFRs), which was involved in the pathogenicity of PDAC [6, 22]. In the present study, we detected the expression of FRS2 in PDAC for the first time. We found that FRS2 was present in 35.06% of PDAC patients. While no significant association was observed between the expression of FRS2 and other clinicopathological factors, it was significantly associated with poor prognosis of PDAC. Our study also revealed that the positive co-expression of GAB2/CRKL/FRS2 was an independent prognostic factor for PDAC.

In the present study, we did not introduce an extra set of training slides that set up the standard for staining intensity as well as the cut-off values for the three adaptor proteins. While allowing for the inclusion of the maximum cases possible in the statistical calculation, the present practice may have brought a certain degree of subjectivity to the interpretation of the IHC results. If GAB2, CRKL, and FRS2 proteins were to be established as new biomarkers for PDAC patients, further validation and standardization processes are compulsory.

## Conclusions

In conclusion, GAB2, CRKL, and FRS2 expression were associated with poor prognosis of PDAC patients. Among them, expression of GAB2 and the co-expression of GAB2/CRKL/FRS2 were independent prognosticators regarding DFS and OS, respectively.

## Abbreviations

CIs: Confidence intervals
CRKL: CRK-like protein
DFS: Disease-free survival
DOK: Downstream of kinase
FFPE: Formalin fixed paraffin embedded
FRS2: Fibroblast growth factor receptor substrate 2
GAB: GRB2-associated binding protein
IRS: Immunoreactive score
IRS: Insulin receptor substrate
OS: Overall survival
PDAC: Pancreatic ductal adenocarcinoma
RTKs: Receptor tyrosine kinases
SHC: Src homology 2-containing protein
